# Seroprevalence of malaria in inhabitants of the urban zone of Antananarivo, Madagascar

**DOI:** 10.1186/1475-2875-5-106

**Published:** 2006-11-10

**Authors:** Olivier Domarle, Romy Razakandrainibe, Emma Rakotomalala, Laurence Jolivet, Rindra Vatosoa Randremanana, Fanjasoa Rakotomanana, Charles Emile Ramarokoto, Jean-Louis Soares, Frédéric Ariey

**Affiliations:** 1 Institut Pasteur de Madagascar, Antananarivo, Madagascar; 2 Ecole Nationale des Sciences Géographiques, Marne la Vallée, France; 3 Institut Pasteur du Cambodge, Phnom Penh, Cambodia

## Abstract

**Background:**

Antananarivo, the capital of Madagascar, is located at an altitude of over 1,200 m. The environment at this altitude is not particularly favourable to malaria transmission, but malaria nonetheless remains a major public health problem. The aim of this study was to evaluate exposure to malaria in the urban population of Antananarivo, by measuring the specific seroprevalence of *Plasmodium falciparum*.

**Methods:**

Serological studies specific for *P. falciparum *were carried out with an indirect fluorescent antibody test (IFAT). In a representative population of Antananarivo, 1,059 healthy volunteers were interviewed and serum samples were taken.

**Results:**

The seroprevalence of IgG+IgA+IgM was 56.1% and that of IgM was 5.9%. The major risk factor associated with a positive IgG+IgA+IgM IFAT was travel outside Antananarivo, whether in the central highlands or on the coast. The abundance of rice fields in certain urban districts was not associated with a higher seroprevalence.

**Conclusion:**

Malaria transmission levels are low in Antananarivo, but seroprevalence is high. Humans come into contact with the parasite primarily when travelling outside the city. Further studies are required to identify indigenous risk factors and intra-city variations more clearly.

## Background

Antananarivo, the capital of Madagascar, is located on hills in the middle of the Central Highlands, at an altitude of between 1,200 and 1,400 m. The city and its surroundings include 1,700,000 inhabitants (2001 census), corresponding to about 10% of the national population. The Antananarivo plain is covered by vast areas of irrigated rice fields, which are potential breeding sites for malaria vectors. Some of the seedier districts in the middle of town have zones that flood easily, favouring mosquito breeding. At the beginning of the 1980s, *Anopheles funestus *reappeared in the Central Highlands of Madagascar [1-3], from which it had disappeared in the 1950s [4]. New epidemic episodes appeared in the middle of the 1980s [5-7], causing several tens of thousands of deaths [8]. Two surveys carried out in Antananarivo in 2003 showed, by biological examinations, that less than 2% of all cases of fever were confirmed to be malaria. About 80% of the confirmed cases had travelled outside the city to areas exposed to malaria in the weeks preceding the survey, the remaining 20% of cases being cases of indigenous malaria due to local transmission [9]. Imported cases outnumber cases of indigenous malaria, and studies have shown only low levels of indigenous malaria transmission. The mosaic nature of the Antananarivo environment exposes the inhabitants of certain zones to the risk of malaria outbreaks.

The history of contact between humans and *Plasmodium falciparum *was examined by measuring seroprevalence in a representative population from the urban zone, to identify factors associated with exposure to malaria.

## Methods

### Population study

The study was conducted using existing serum samples from inhabitants of Antananarivo who had been enrolled in February 2004 for a study on hepatitis C. Volunteers were selected by cluster sampling with two degrees of freedom, based on the cluster sampling method used in immunisation coverage programmes [10]. This mode of random and cluster sampling yielded a cohort of subjects representative of the population of Antananarivo. Seventy clusters of 13 to 18 people were selected for this investigation, to maximise the effect of clusters and displacements (as described in [10]). Once informed consent had been obtained, the subjects were interviewed and serum samples were obtained from blood collected in dry tubes for biological analyses. A questionnaire was designed to evaluate the influence of associated factors: the number of journeys involving at least one night outside the city in the last six months, number of antimalarial treatments in the last six months and level of schooling (to evaluate socio-economic status).

### Screening for antibodies specific for *P. falciparum*

For antibody screening, the indirect fluorescent antibody test (IFAT) was used [11,12]. Slides were coated with the Palo Alto strain of *P. falciparum *from continuous *in vitro *culture. Serum samples, at successive dilutions between 1/64 and 1/4,096, were incubated with the parasites on the slide. A reaction with the 1/64 dilution is generally regarded as the threshold for a positive reaction [13]. The antibody-antigen complex was detected using sheep F(ab')2 anti-human-IgM conjugated with fluorescein isothiocyanate (FITC), or sheep (H+L) anti-human-IgG+IgA+IgM conjugated with FITC (Bio-Rad, France). All incubations were performed at room temperature, in a dark, humid chamber, for 45 minutes. Between incubations, slides were washed three times in phosphate-buffered saline. All tests were done in parallel with seropositive and seronegative control samples from the serum libraries of the laboratory. Slides were examined with an epifluorescence microscope. Antibody concentration was determined semi-quantitatively, by noting the highest dilution factor at which the serum gave fluorescent spots on incubation with the parasites.

Data were analysed in two different ways. A dichotomous classification of samples as seronegative or seropositive was used for analyses of seroprevalence and statistical analysis. To take into account the range of antibody concentrations and transformed individual into collective data, subjects were assigning into categories, according to common criteria, and the geometric mean of antibody rates (GMAR) were calculated according to the following formula (n_x _is the number of subjects positive at the dilution rate of x; d_x _is the dilution factor for the dilution rate of x, for example, the d_x _value for 1/64 is 64; for sera negative for all dilution rates, Log(d_x_) = 1):

GMAR=10∑nx⋅Log(dx)∑nx
 MathType@MTEF@5@5@+=feaafiart1ev1aaatCvAUfKttLearuWrP9MDH5MBPbIqV92AaeXatLxBI9gBaebbnrfifHhDYfgasaacH8akY=wiFfYdH8Gipec8Eeeu0xXdbba9frFj0=OqFfea0dXdd9vqai=hGuQ8kuc9pgc9s8qqaq=dirpe0xb9q8qiLsFr0=vr0=vr0dc8meaabaqaciaacaGaaeqabaqabeGadaaakeaacqqGhbWrcqqGnbqtcqqGbbqqcqqGsbGucqGH9aqpcqaIXaqmcqaIWaamdaahaaWcbeqaamaalaaabaWaaabqaeaacqqGUbGBdaWgaaadbaGaeeiEaGhabeaaliabgwSixlabbYeamjabb+gaVjabbEgaNjabcIcaOiabbsgaKnaaBaaameaacqqG4baEaeqaaSGaeiykaKcameqabeGdcqGHris5aaWcbaWaaabqaeaacqqGUbGBdaWgaaadbaGaeeiEaGhabeaaaeqabeGdcqGHris5aaaaaaaaaa@494E@

### Geographical analysis

Rice fields are thought to be the most favourable environment for the development of malaria vectors in the ecosystems of Antananarivo [1-3,7]. Two types of image were used to identify rice fields [14]. The Landsat Enhanced Thematic Mapper (ETM+) image was acquired on May 2000 and radar images (Envisat) were acquired in January and July 2004. Rice fields differ from other types of vegetation in continually changing state over the course of the year.

Landsat images were enhanced by creating a colour composite image from the seven spectral bands of the original image, to make it easier to recognise objects on the ground. This was achieved by assigning bands to one of the three channels (blue, red, green). This makes it possible to calibrate various responses on the image: differences in colour and texture are associated with different classes of land cover. Principal component analysis (PCA) was then used to create neo-channels with more than 95% of information in the first axis, PCA1 [15,16].

Two periods of field work were carried out. The first period was used to identify training sites and test sites. The GPS (Gerographical Positionning System) co-ordinates of the rice fields visited during these field studies are given. The second period of field work was used to check that the features on the ground corresponded to the images obtained. The training sites were digitised and supervised classification by the maximum likelihood method was used to generate a map of rice fields. The accuracy of this classification accuracy was estimated, using the Kappa coefficient [17]. The test sites were used to produce a confusion matrix, for assessing the overall accuracy of the land cover classification map.

Radar images were calibrated before absolute georeferencing. Image enhancement was designed to reduce speckling but to preserve accuracy. Adaptive filters were applied to ensure the highest possible image quality. Landscape changes between January and July were compared by visual image interpretation and backscatter coefficient analysis [18,19]. A rice field index was calculated by dividing the area under rice by the area of the "Fokontany", the basic administrative district in the city [20].

### Statistical analysis

Values of *p *< 0.05 were considered significant in all statistical analyses. Univariate analysis was carried out with Pearson's chi-squared test as implemented in EPI-info software. Chi^2^ tests were used to compare qualitative variables. Multivariate analysis was carried out by means of backward stepwise logistic regression, using SPSS^® ^software (version 11.5). The dependent variable was positive IgG+IgA+IgM tests for malaria. Variables with *p *values below 0.2 in univariate analysis were introduced into the model and kept constant during the first step.

## Results

### Characteristics of the cohort

The study was carried out on 1,059 subjects (70 clusters of 13 to 18 subjects) with a male/female sex ratio of 0.59. The mean age of the subjects was 29.56 ± 17.66 years. Most (89.6%) had settled in Antananarivo in the four years before the survey. The investigators asked the subjects how many times they had spent at least one night outside of Antananarivo in the previous six months: 28.14% had spent at least one night outside the city on journeys to the Central Highlands (17.75%) or coastal zones (10.39%). Patients were asked about the frequency of malaria treatment during the previous six months: 82.63% had no malaria treatment, 12.65% had one course of malaria treatment and 4.72% had more than one course of malaria treatment. Socio-economic status was estimated, using a classification based on education level: no schooling (4.63%), primary school level (35.41%), secondary school level (49.01%) and post-secondary studies (10.95%).

### Rice field index

**Figure 1:**
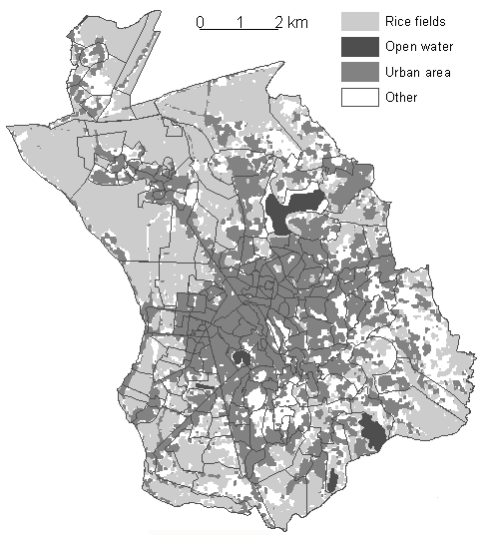
**Map of Antananarivo with its 192 districts ("fonkontany" in Malagasy) and rice fields located by satellite images**. A comparison of satellite images in different seasons made it possible to locate the rice fields (in light grey) and to differentiate them from the other types of vegetation (in white: bare soil, vegetation, other crops). Districts are delimited as indicated on the map. For each district, rice field index was calculated by dividing the area under rice by the area of the entire district.

The rice field index, calculated from satellite images, was used to classify city districts according to the percentage of the surface area suitable for potential anopheline mosquito breeding sites (Figure 1). Its value varied from 0% to 86% (median = 4.76%; first quartile 0%; third quartile = 26.99%).

### Serology

Table 1 shows the results for IgG+IgA+IgM testing. The seroprevalence was 56.10%. The seroprevalence in men was significantly higher than that in women (60.1% and 53.7%, respectively; chi^2 ^test, *p *= 0.044). The serological prevalence of IgG+IgA+IgM increased significantly with age (under or over 15 years of age, chi^2 ^test, *p *< 0.0001). Univariate analysis (Table 1) showed significant associations between IgG+IgA+IgM-positive IFAT, travel outside the city, and rice field index. Travels outside the city were associated with a higher seroprevalence, and the GMAR of those who travelled (coast or Central Highlands) was higher than that of subjects who did not travel (GMAR coast group = 29.3; GMAR Central Highlands group = 30.2; GMAR for the group that did not travel = 16.1). Seroprevalence was analysed as a function of rice field index. A significant difference was observed between the subjects of the third quartile (rice field index > 26.99%) and the other subjects, with people living in districts with a high rice field index having a lower IgG+IgA+IgM prevalence. Sex, socio-economic level, history of travel outside Antananarivo and rice field index were included in a logistic regression model. Multivariate analysis confirmed that age, travelling outside Antananarivo and rice field index were significantly associated with the presence of IgG+IgA+IgM (Table 2). The seroprevalence of IgM was about 5.85%, and was not associated with other variables.

**Table 1:** Factors associated with IgG+IgA+IgM seroprevalence

			***P. falciparum *IgG+IgA+IgM**		
		**n**	**negative**	**positive**	***P***
**Entire cohort**		1,059	43.9%	56.1%	
Sex	Sex ratio (male/female) = 0.59				
	Male	391	14.7%	22.2%	0.044
	Female	668	29.2%	33.9%	
					
Age	Mean age = 29.56 ± 17.66 years				
	< 15 years	251	12.9%	10.8%	< 0.0001
	≥ 15 years	808	31.0%	45.3%	
					
Social class	No schooling	49	2.2%	2.5%	NS
	Primary school level	375	16.8%	18.6%	
	Secondary school level	519	20.3%	28.7%	
	Post-secondary studies	116	4.6%	6.3%	
					
Travel outside city (previous 6 months)	No travel	761	33.4%	38.4%	0.021
	Travel to Central Highlands	188	6.4%	11.3%	
	Travel to the coast	110	4.1%	6.3%	
					
Malaria episodes (previous 6 months)	0 declared episodes, no treatment	875	36.5%	46.2%	NS
	1 declared episode with treatment	134	5.7%	7.0%	
	More than 1 episode	50	1.8%	2.9%	
					
Rice field index	Lower than 26.98%	785	29.6%	44.5%	< 0.0001
	Higher than 26.99% (last quartile)	274	14.3%	11.6%	

**Subjects living in the city for more than 4 years and had not travelled outside the city in the previous 6 months**		690	46.5%	53.5%	
Sex	Sex ratio (male/female) = 0.49				
	Male	226	14.1%	18.7%	NS
	Female	464	32.5%	34.8%	
					
Age	Mean age = 30.25 ± 18.07 years				
	< 15 years	165	13.3%	10.6%	0.006
	≥ 15 years	525	33.2%	42.9%	
					
Rice field index	Lower than 26.98%	512	32.3%	41.9%	0.008
	Higher than 26.99% (last quartile)	178	14.2%	11.6%	

**Table 2:** Multivariable analysis of the variables associated with IgG+IgA+IgM seropositivity

	**Coef.**	**CI 95%**	***P***
**Entire cohort. n = 1,059**			
Age (< 15 vs ≥ 15 years)	1.012	[1.005 1.019]	0.001
Travel outside city (previous 6 months; no travel vs travel)	1.360	[1.026 1.801]	0.032
Rice field index (low vs high (last quartile))	0.554	[0.424 0.725]	< 0.001

**Subjects living in the city for more than 4 years and had not travelled outside the city in the previous 6 months, n = 690**			
Age (< 15 vs ≥ 15 years)	1.013	[1.004 1.022]	0.004
Rice field index (low vs high (last quartile))	0.583	[0.419 0.810]	0.001

## Discussion

Sampling was designed to ensure that the cohort of subjects enrolled was representative of the population of Antananarivo. However, the male/female sex ratio was 0.59. This may be explained by the logistics of sample collection, which occurred when men were at work. Men travelled outside Antananarivo significantly more frequently than women (data not shown), potentially accounting for the significantly larger number of male carriers of *P. falciparum *antibodies. As expected, age was significantly related to an increase in the frequency of positive IFAT results. This relationship is accounted for by the increase in the cumulative risk of human/parasite contact with age, and by travelling outside the city being more common in subjects over the age of 15 years than in younger subjects (chi-squared test, *p *< 0.05, data not shown).

Rice fields are thought to be the principal location of anopheline mosquito breeding sites in Antananarivo [1-3,7]. However, data show an inverse relationship between rice field index and seroprevalence for IgG+IgA+IgM. IgG+IgA+IgM rates are in major part determined by IgG. It is difficult to estimate the relationship between the rates of IgG and the intensity and history of human-parasite contact, particularly if this contact is regular. It is commonly thought that IgG can be detected several years after contact with the parasite. However, Phase I trials of malaria vaccines have shown that most subjects become seronegative within six to eight months of the last injection [21-23], or remains detectable 12 months after immunization in only a minority of subjects [24]. In this present study, when data were analysed with subjects limited to those who had lived in Antananarivo for more than four years and who had not travelled outside the city in the six months before the investigation, the inverse relationship between rice field index and IgG+IgA+IgM prevalence in IFAT persisted. It is possible that travelling outside the city more than six months before the study may still have had a very strong effect on IgG rates.

The rice field index calculated for each district did not take into account the distance between mosquito breeding sites and dwellings. The level of transmission, the risk of malaria and seroprevalence may depend on the distance between mosquito breeding sites [25-27], rather than the total surface area covered by such sites.

Subjects with a higher level of education (secondary school level or higher) were significantly more likely to have travelled outside the city in the previous six months than less educated subjects (no schooling or primary school only). However, the least educated subjects lived in the districts with the highest rice field index (data not shown), when those with the highest social standing lived in the most salubrious districts, with a low rice field index, but frequently travelled outside the city, resulting in higher risk of malaria in this group. However, multivariate analysis identified no significant influence of educational level on the relationship between rice field index and IgG+IgA+IgM prevalence. A recent study in Accra and Kumasi, Ghana, showed that low socio-economic status was associated with a high prevalence of malaria [28]. Malaria transmission was higher in Accra than in Antananarivo. Local malaria transmission in Antananarivo seems to be a minor risk that it may be difficult to distinguish. Other studies have reported a 'paddies paradox' based on parasitological studies in different malaria transmission areas [29-34]. However, the context is very different for Antananarivo, with its urban zone (unfavourable for malaria vectors) and a mosaic of rice fields (more suitable for the breeding of *Anopheles *than the urban environment). The causal factors are therefore unlikely to be the same.

Unexpectedly, the study showed that a high density of potential larval habitats was associated with a lower prevalence of specific *P. falciparum *antibodies. However, this relationship was skewed by i) journeys outside the city in the years preceding this study (frequency and duration), ii) differences in the frequency of journeys outside the city between social classes, iii) the pattern of settlement in the city, which depends on socio-economic status and sanitary conditions, iv) the fact that the rice field index of the district did not take into account the distance between the larval habitat and the dwelling places of the enrolled subjects.

Like other studies [9,35-38], this study showed that travels outside the city were a major risk factor for malaria. However, this study also reveals the peculiarity of the urban environment for malaria. Urban malaria occurs in a particular context, and the rules governing its occurrence may differ from those described for the rural environment [39]. Nowadays, more than half the African population lives in cities. Better knowledge of the urban context is necessary to optimise strategies to fight malaria.

## Authors' contributions

FA initiated this project and designed the study whilst at Institut Pasteur de Madagascar. J-LS and CER were the investigators in the hepatitis C survey leading to this study of malaria. They were responsible for statistical analysis of the data. FR, RVR and LJ carried out all the geographical parts of the study. RR and ER carried out the IFAT tests. OD was the principal investigator. He analysed and interpreted the data and wrote the manuscript. All the authors have revised and approved the final manuscript.
